# Subgroup detection in genotype data using invariant coordinate selection

**DOI:** 10.1186/s12859-017-1589-9

**Published:** 2017-03-16

**Authors:** Daniel Fischer, Mervi Honkatukia, Maria Tuiskula-Haavisto, Klaus Nordhausen, David Cavero, Rudolf Preisinger, Johanna Vilkki

**Affiliations:** 1 Natural Resources Institute Finland (LUKE), Myllytie 1, Jokioinen, Finland; 2 Department of Mathematics and Statistics, University of Turku, Turku, Finland; 3 Lohmann Tierzucht GmbH, Am Seedeich 9-11, Cuxhaven, 27454 Germany; 4 University of Tampere, School of Health Sciences, Medisiinarinkatu 3, Tampere, 33014 Finland

**Keywords:** ICS, PCA, Genotype data, Classification, Dimension reduction

## Abstract

**Background:**

The current gold standard in dimension reduction methods for high-throughput genotype data is the Principle Component Analysis (PCA). The presence of PCA is so dominant, that other methods usually cannot be found in the analyst’s toolbox and hence are only rarely applied.

**Results:**

We present a modern dimension reduction method called ’Invariant Coordinate Selection’ (ICS) and its application to high-throughput genotype data. The more commonly known Independent Component Analysis (ICA) is in this framework just a special case of ICS. We use ICS on both, a simulated and a real dataset to demonstrate first some deficiencies of PCA and how ICS is capable to recover the correct subgroups within the simulated data. Second, we apply the ICS method on a chicken dataset and also detect there two subgroups. These subgroups are then further investigated with respect to their genotype to provide further evidence of the biological relevance of the detected subgroup division. Further, we compare the performance of ICS also to five other popular dimension reduction methods.

**Conclusion:**

The ICS method was able to detect subgroups in data where the PCA fails to detect anything. Hence, we promote the application of ICS to high-throughput genotype data in addition to the established PCA. Especially in statistical programming environments like e.g. R, its application does not add any computational burden to the analysis pipeline.

**Electronic supplementary material:**

The online version of this article (doi:10.1186/s12859-017-1589-9) contains supplementary material, which is available to authorized users.

## Background

The fast progress in analyzing variations in the genome by deep sequencing has led to a plethora of high density genotyping arrays in many livestock species. Thereby also, the amount of single nucleotide polymorphism (SNP) data that is available for analyzing the genetic relationships between different samples is constantly growing. One common approach to handle this type of data and to identify e.g. subpopulations, is the application of dimension reduction methods such as Principle Component Analysis (PCA). Currently PCA is established to be the standard approach in clustering genotype data, see e.g. [1]. However, as we will demonstrate with an simulation example, there are drawbacks and pitfalls in the PCA approach. In a PCA, the principle components are ordered according to the variance they explain, but there is no theoretical justification that the component with the largest explained variance also contains the information required, e.g. to separate subgroups within the data. A vivid counterexample is a large hamburger. If it is large enough, the component with the largest variation goes through the diameter of the burger, but to separate the subgroups, one would need a direction from bottom to top. That means, even in this simple three-dimensional case the interesting component would be only the second one. Hence, the interesting components might explain only a small fraction of the variance and consequently are easily missed by checking only the few first or last components. For an overview and a more theoretical background on the application of PCA in genotype data, see [2] or [3].

Other dimension reduction methods, such as Invariant Coordinate Selection (ICS), are not commonly applied to genomic data. ICS is a modern multivariate method originally introduced as generalized PCA in [4], but then established as ICS in the seminal paper [5] to avoid a name mismatch with a different generalized PCA approach, see e.g. [6].

The basic idea of ICS is to use two different scatter matrices and to compare how they differ. Different choices of scatter matrices lead then to different applications of ICS. Currently, ICS has been e.g. applied to near-real time retrieval of low stratiform cloud coverage [7]. Further, ICS was used to enhance the discrimination between snow and ice clouds and detection of broken, thin clouds [8] and also for studies of developmental canalization and the identification of divergent and stabilizing selection [9].

To discuss possible problems with PCA, we will first present a basic counterexample to show that PCA does not necessarily identify cluster structures in a dataset and after that apply PCA and ICS on a real example genotype dataset. Further, for both datasets we will compare the two methods also with other methods used by the Bioinformatics community. For that we apply also t-distributed Stochastic Neighbor Embedding (t-SNE) [10], Isomap [11], Locally Linear Embedding (LLE) [12], kernel PCA (kPCA) [13] and Diffusion Maps (DM) [14] to the simulated and the real data. For completeness, we also check the performance of a Linear Discriminant Analysis (LDA) for the simulation example.

## Methods

### Simulated data

First we simulated a dataset as an example that PCA is not always capable of detecting clusters in high-dimensional data. Consider three 10-variate normal populations with *N*_10_(***μ***_*i*_,***Σ***), where

***μ***_1_=(−*μ* ^∗^,*μ* ^∗^,0,0,0,0,0,0,0,0)^⊤^,

***μ***_2_=(*μ* ^∗^,0,0,0,0,0,0,0,0,0)^⊤^ and

***μ***_3_=(0,−*μ* ^∗^,0,0,0,0,0,0,0,0)^⊤^ and

$\boldsymbol {\Sigma }=\text {diag}\left (\frac {1}{5},\frac {1}{5},1,1,1,2,2,2,2,2\right)$ with *μ* ^∗^=2. From each population we simulated then 100 samples. In order to hide the clearly visible subpopulations, we further rotated the simulated observations with a random orthogonal matrix. Note that the rotation has no impact on the performance of PCA as the method is rotation invariant.

Additional file 1: Figure S1 shows the simulated data before the rotation and Additional file 1: Figure S2 the data after it. In the latter one, the groups are clearly not visible anymore.

### Chicken data

The high-density genotype data consists of 749 chicken from 4 generations. The last generation is the largest group with 603 samples. The other generations contain 50, 46 and 50 samples. The data consists of sequence based variation data from 7 genomic regions, covering approx. 35% of the genome. The regions have been preselected based on previous studies as containing loci affecting egg-quality traits, see [15] and [16]. As reference genome we used galgal4.

In total there were 157,528 genotypes measured in those regions. See Additional file 1: Figure S3 for the locations of the used regions on the chicken reference genome.

In addition to the genotype data, also a set of 15 different breeding values was available for all chicken. These were, besides others, egg production in period 3 to 7, egg production in period 9 to 12 and feed intake. We use this data as real data example and will follow up on the biological findings only for one detected subgroup in order to keep the focus on the method.

### Invariant coordinate selection

To explain ICS we need first to introduce the concept of scatter matrix. For a *p*-variate random vector **x** any *p*×*p* matrix-valued estimator **S**(**x**) is called a scatter matrix if it affine equivariant in the sense that

$$\mathbf{S}(\mathbf{A}\mathbf{x} + \mathbf{b}) = \mathbf{A}\mathbf{S}(\mathbf{x}) \mathbf{A}^{\top}, $$

for any full-rank *p*×*p* matrix **A** and any *p*-variate vector **b**. Clearly the regular covariance matrix COV is a scatter matrix. But especially in the robust statistics literature many other scatter matrices were introduced. For more details about how scatter matrices generalize the covariance matrix and many related references see [17]. A scatter matrix we will use later is the so-called scatter matrix of fourth moments

$$\text{COV}_{4}(\mathbf{x}) = \frac{1}{p+2} \mathbf{E}\left(r^{2} (\mathbf{x}- \mathbf{E}(\mathbf{x}))(\mathbf{x}-\mathbf{E}(\mathbf{x}))^{\top}\right), $$

where *r*=||COV(**x**)^−1/2^(**x**−**E**(**x**))|| and ||·|| denotes the Frobenius norm.

The main idea of ICS is to compare two different scatter matrices **S**_1_(**x**) and **S**_2_(**x**) by solving the following eigenvector-eigenvalue problem

$$\mathbf{S}_{1}^{-1}(\mathbf{x}) \mathbf{S}_{2}(\mathbf{x}) \mathbf{B}^{\top}(\mathbf{x}) = \mathbf{B}^{\top}(\mathbf{x}) \mathbf{D}(\mathbf{x}), $$

where **D**(**x**) is then the diagonal matrix containing the *p* eigenvalues of $\mathbf {S}_{1}^{-1}(\mathbf x) \mathbf {S}_{2}(\mathbf {x})$ in decreasing order. The rows of **B**(**x**) contain then the corresponding eigenvectors. For convenience of notation we will denote from now on **S**_1_(**x**)=**S**_1_, **S**_2_(**x**)=**S**_2_, **B**(**x**)=**B** and **D**(**x**)=**D**.

The ICS equation above can be seen as the problem of jointly diagonalizing the two scatter matrices, i.e. find **B** and **D** such that

$$\mathbf{B}\mathbf{S}_{1} \mathbf{B}^{\top} = \mathbf{I}_{p} \quad \text{and} \quad \mathbf{B}\mathbf{S}_{2} \mathbf{B}^{\top} = \mathbf{D}. $$

An interpretation can then be given as follows. First **S**_1_ is used to whiten the data, i.e. uncorrelate the variables and standardize the scales. Then perform on the whitened data PCA using **S**_2_. Therefore the idea is to see if **S**_2_ finds still some interesting structure after removing second order information as measured by **S**_1_.

The transformation **B**(**x**)**x** yields then an invariant coordinate system in the sense that

$$\mathbf{B}(\mathbf{x})\mathbf{x} = \mathbf{B}(\mathbf{A}\mathbf{x}) (\mathbf{A}\mathbf{x}), $$

where equality holds up to marginal signs for any full rank *p*×*p* matrix **A**. The new vector **z**=**B**(**x**)**x** is then usually referred to as the invariant coordinates.

The univariate concept of kurtosis can be seen as the ratio of two (standardized) scale measures and similarly $\mathbf {S}_{1}^{-1}\mathbf {S}_{2}$ can hence be seen as a multivariate extension of this concept. Therefore the eigenvalues contained in **D** can be interpreted as generalized kurtosis values as measured by **S**_1_ and **S**_2_. In the special case of **S**_1_=COV and **S**_2_=COV_4_ it can be shown that the diagonal elements in **D** are a linear function of the classical measures of kurtosis of the components in **z** [18].

And for example when searching clusters it is well-known that large clusters can be found often in directions with small kurtosis and outliers and small clusters in directions with large kurtosis. This means that invariant coordinates are very suitable for searching for groups as the components are ordered according to their (generalized) kurtosis. As actually [5] show, in the context of mixtures of elliptical distributions with proportional scatter matrices, ICS finds Fisher’s linear discriminant subspace without knowing the group memberships. Hence, when using ICS for exploratory data analysis usually most attention is paid to the components with extreme generalized kurtosis values, like for example the first 3–5 and last 3–5 components. For more details about ICS see [4, 5, 18, 19].

As practical considerations we would however like to point out that there is no general best combination of scatter matrices and the performance might depend on the choice of *S*_1_ and *S*_2_. The choice *S*_1_=COV and *S*_2_=COV_4_ is however well-established and for example also a solution to the independent component problem (ICA) if **x** follows it (see eg [20] for further details). ICA has been applied in the context of genetic data e.g. in [21].

Furthermore, ICS is however currently limited to the case when *p*<*n*−1 as otherwise scatter matrices are always proportional to each other, see [22] for details. Therefore if *p*≥*n*−1, then one can for example first perform dimension reduction using PCA, resulting in a *n*×*n* matrix where the *n*-th eigenvalue is zero. Then ICS is only applied to a subspace which is known to have variation and is of smaller dimension than *n*−1. This is for example standard practice in many multivariate methods which are limited to the *p*≥*n*−1 case, like for example the high-dimensional noisy ICA approaches [23].

### Distance measure, distance groups and statistical testing

For the simulated data the classification decision based on the scatterplot matrices from PCA and ICS was done by applying a k-means algorithm to the desired components. The classification results of the different dimension reduction methods were then evaluated using the adjusted Rand index [24]. In the real data example, the classification decision was done by visual inspection of the figures.

In order to calculate the genetic distance of two different groups in a region of interest, we followed a basic approach. Assuming two subpopulations *A* and *B* have been identified in the data, we determined first at each loci *l*=1,2,… the most common genotypes for both groups and denote these $\tilde {G}_{A,l}$ respective $\tilde {G}_{B,l}$. Then, we compared if these genotypes match between the two groups, by setting *G*_*l*_=1, if $\tilde {G}_{A,l} = \tilde {G}_{B,l}$ and 0 else. Afterwards we calculated a moving average of length 1000 across the data and calculated in each window the average level of agreement. Let *W*=*w*_1_,*w*_2_,… be the set of all windows of length 1000 with *w*_1_=*l*_1_,…,*l*_1000_,*w*_2_=*l*_2_,…,*l*_1001_, the average level of agreement in window *i* is then $\bar {x}_{w_{i}}=\sum _{\forall l \in w_{i}} G_{l} / 1000$. For the sake of simplicity, we calculated the moving average also across chromosomal borders.

For all windows *w*_*i*_ with level of agreement between two subpopulations $\bar {x}_{w_{i}} \leq 0.4$, the individual distance of each individuum in the one group was calculated to the average of the other group. For that, we use again the most common genotype for each loci in the subpopulation coded as 0,1,2 and then we calculated the Manhattan distance of each individuum from the standard population to that.

Testing for differences in the breeding values between the two subpopulations has been done by applying a two-sided Mann-Whitney test. Significant breeding values (*p*-value ≤0.05) are further investigated with a directional test, as proposed by [25] and implemented in the R-package *gMWT* [26]. The individual distance measure of the chicken from the main population to the subpopulation showed three types of chicken, those which are genetically close (*c*), those that are medium (*m*) and those that are far (*f*) away from the subpopulation. Let *F*_*p*,*c*_, *F*_*p*,*m*_ and *F*_*p*,*f*_ be the distributions of the three groups for a given phenotype *p*, we have then the following two testing problems in mind

$$H_{0}: F_{p,c} = F_{p,m} = F_{p,f} \quad \text{vs} \quad H_{1}: F_{p,c} \preceq_{st} F_{p,m} \preceq_{st} F_{p,f} $$

or

$$H_{0}: F_{p,c} = F_{p,m} = F_{p,f} \quad \text{vs} \quad H_{2}: F_{p,f} \preceq_{st} F_{p,m} \preceq_{st} F_{p,c} $$

with ≼_*st*_ being the stochastical ordering of the two distributions. Two distributions *F*_1_ and *F*_2_ are stochastically ordered, if *F*_1_(*x*)≥*F*_2_(*x*) ∀*x*∈*R* and we write *F*_1_≼_*st*_ *F*_2_. These directional hypotheses have been used to test for a directional relationship between the similarity group and the different phenotypes.

## Results

To evaluate the performance of the different dimension reduction methods to unravel the original cluster structure, we first clustered the plain simulated data using k-means with the constrain of three classes (*k*=3). For the classification result, we then calculated the adjusted Rand Index for the 3×3 table between the original class labels and the result of the k-means clustering. Next, we performed a PCA, followed again by a k-means clustering using the first two components for classification. Also for this classification result table we calculated the adjusted Rand index. Then we applied ICS onto the dataset and calculated in the same way again the Rand index for the k-means applied to the last two components. To compare the results to other popular dimension reduction methods, we applied also t-SNE, Isomap, LLE, kPCA and DM to the simulation data and calculated the corresponding Rand indices. Further, we searched with each dimension reduction method the same dimensionality, that was *d*=2 for the simulated and *d*=7 for the real chicken data.

**Fig. 1:**
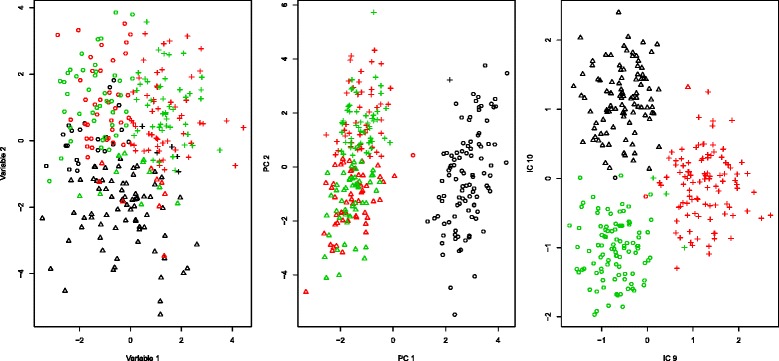
Cluster labels of the k-means clustering for mixed data (*left*), the first two principle components (*middle*) and the last two ICS components (*right*). The true class labels are colored accordingly and the k-means classification is represented with different symbols

The Rand index for the clustering using the original data is 0.20, the index for PCA is 0.48 and for ICS it is 0.94. In other words, the k-means clustering applied to the raw data does not detect any of the original groups and the PCA only detects two groups, but mixes the second and third one. The ICS method, however, recovers the original cluster structure to a large extent, indicated by an adjusted Rand index of nearly one. See also Fig. 1 that visualizes the cluster labels in the projected datasets for the k-means classifications applied to the different methods.

The results for the other dimension reduction methods were rather weak. Whereas the t-SNE method was almost as good as the ICS (Rand-index 0.93), the four others clearly were outperformed by these two mothods. Isomap had a Rand index of 0.71, LLE had a value of 0.48, DM had also only 0.50 and the kPCA method had with 0.42 even a value smaller than the PCA had. That means, none of these methods was able to fully recover the original data. The corresponding Figures S4–S11 can be found in the Additional file 1. To calculate the t-SNE we used the R-Package tsne [27], Isomap is implemented in the R-Package RDRToolbox [28] and LLE in lle [29]. For kPCA we used the kernmap package [30] and for DM the destiny package [31].

The lda function applied to the simulation data resulted in an error-free separation of the data and had consequently a Rand index of 1. However, the ICS method is with 0.94 not too far away from that optimum. In absolute numbers, 6 out of 300 observations were mislabeled using the ICS function. LDA cannot be applied to the real example data, as the identification of subgroups is done without any prior knowledge and as such supervised methods like LDA cannot be applied to the problem.

**Fig. 2:**
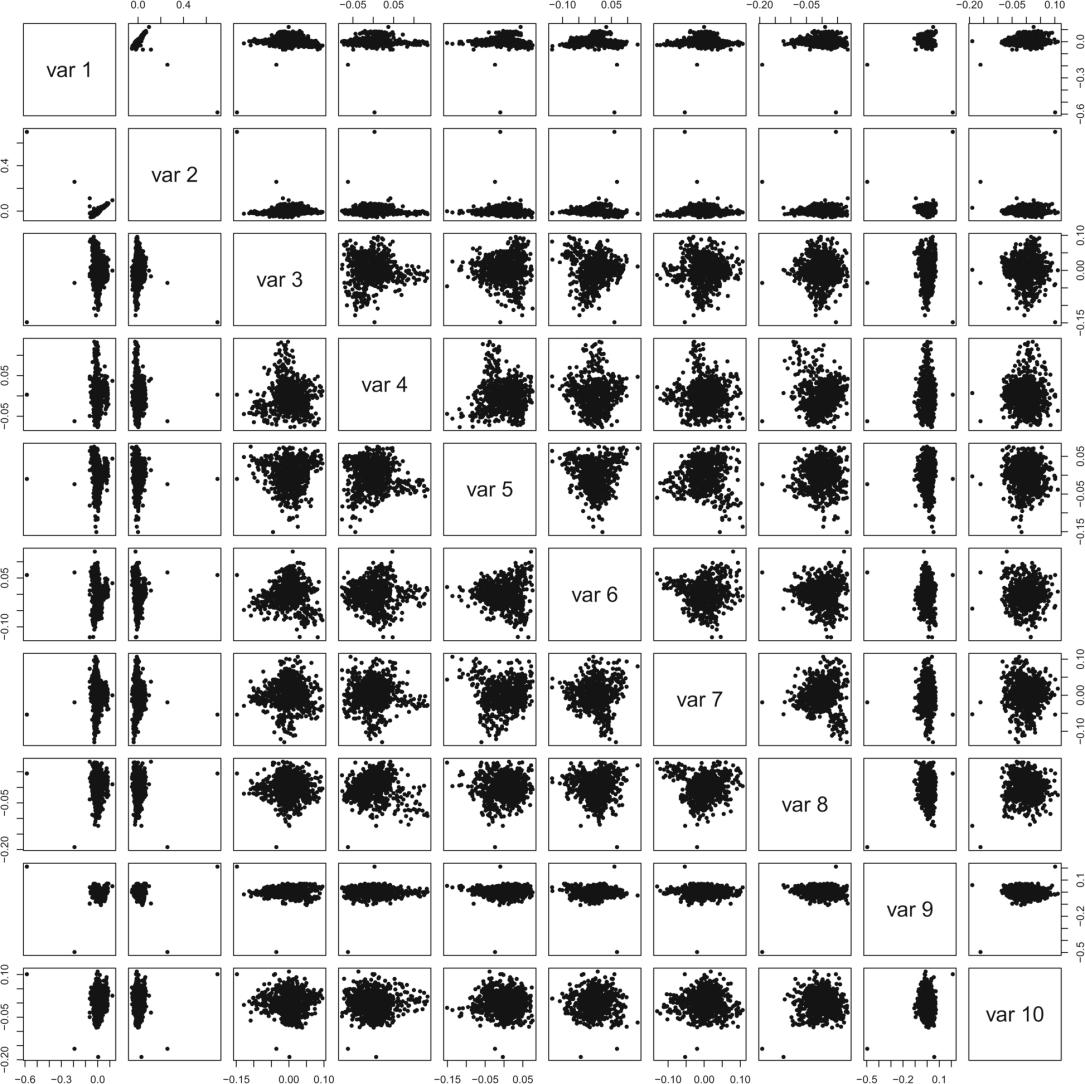
Scatterplot matrix of the PCA analysis. No particular subgroup could be identified. The first component detects only two outlying observations

To analyze the real chicken data using PCA, we applied the snpgdsPCA function of the SNPRelate [32] R-package to it. Figure 2 shows the scatter plot matrix of the ten first components, but no particular subgroup could be identified. The PCA identifies only two strongly deviating individuals. Next we determined the number of eigenvectors that account for a total variance of 80%.

**Fig. 3:**
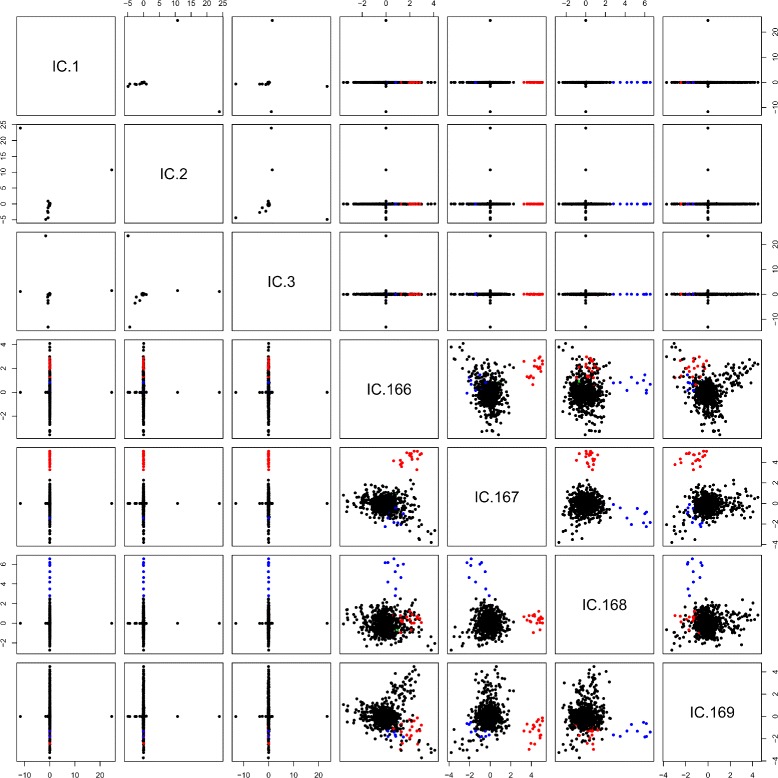
Scatterplot matrix of the ICS analysis. Clear subgroups could be identified in component 167 and 168. All members of the subgroup 167 have the same father, but two different mothers, indicated by *red*(*n*=19) and *green* (*n*=1). Another subgroup could be identified in component 168 (*blue*, *n*=10)

We plugged the corresponding matrix with the first 169 eigenvectors from the eigen-decomposition of the PCA into the ics function of the ICS [19] R-package. We applied the ics function using the regular covariance matrix and the covariance matrix of forth order moments (default), as described above. By using this method we could clearly identify two subgroups in the last components of the ICS as well as deviating individuals in the first components. One subpopulation is separated by the antepenultimate component (Number 167). This subpopulation of 20 individuals is marked in red and green in the scatterplot matrix of the ICS components, see Fig. 3. Further, we could also identify another possible subgroup of size 10 by projecting the data onto the penultimate component (Number 168), indicated in blue. We do not follow up on the individual outliers identified in the first components as the current goal was subgroup detection.

Before analyzing the phenotypical particularities of the identified subgroup, we also test the performance of the other dimension reduction methods on the real chicken data. Here, kPCA and LLE are able to identify the same clusters as ICS does, but t-SNE and Isomap fail to identify any clear cluster structures. In case of t-SNE we tried both *k*=7 and *k*=2, but in neither case any obvious subgroup could be identifed. Diffusion map, however, apparently identifies another subgroup. The corresponding scatterplot matrices can also be found in the Additional file 1. We used the default settings and protocols as provided by the different packages. That means, e.g. for LLE we calculated the optimal number of neighbors as 17.

Members of the red subgroup, identified by the ICS method are all offsprings from the same father and mainly from the same mother. From the 20 members of the subgroup only one individual (indicated by green) has a different mother. The subpopulation indicated in blue is also formed by a family. Seven chicken from this population have the same father and mother. Further, the father of those 7 chicken can also be found in this group.

A region of approximate length 4Mb (Chr2:70,348,413-74,448,870), containing 1340 SNPs was identified by calculating the genetic similarity between the deviating (red) family and the remaining population. The genetic similarity was calculated with a moving average using windows of size 1 kb. Areas, where the average level of agreement drops below 0.4 are considered to be the major cause for the difference between the divergent red family and the main population. Additional file 1: Figure S12 shows the level of agreement across the considered chromosomal regions. Also for the blue subpopulation we could identify in a candidate region a similar way.

Next, we calculated for each chicken within the main population the Manhattan distance between the mode genotype values of the deviating red family in the region of interest and the individual genotypes. There we could clearly identify three subgroups within the main population, see Additional file 1: Figure S13. We denote those subgroups as *close*, *intermediate* and *far*.

When breeding values of 15 production values were compared between the red subpopulation and the main population, significant differences were seen in 10 traits (The two-sided Mann-Whitney test was significant at level *α*=0.05). These were then tested further using a generalized Mann-Whitney test for directional alternatives. This means, we tested for a directional trend of the phenotypes with respect to the close, the intermediate and the far group.

**Fig. 4:**
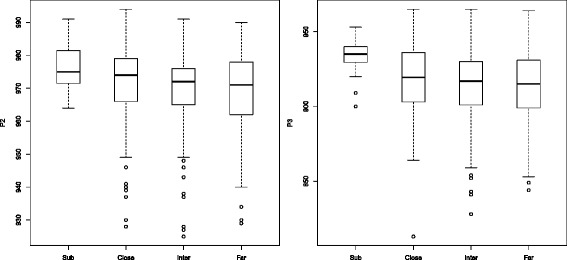
Boxplot of production values P2 (*left*) and P3(*right*). A clear directional relationship between the subpopulation and the three distance groups close, medium and far. In both production periods have chickens that are in the identified region closer to the subpopulation also higher production values

For six breeding values a directional relationship in the main population could also be verified. Especially the production values followed a directional order, see the corresponding boxplots in Fig. 4. In details that means that the red subgroup had a significant higher egg production compared to the main group and within the main group the chicken that are genetically closer to the subgroup in an identified region also had a higher production compared to those that are genetically further away. However, the increased production values occurred with a higher feed intake.

## Discussion

We applied the modern dimension reduction method ICS to a simulation example and compared it to the commonly used PCA method to visualize some deficiency of the PCA approach. Further, we applied the other, modern dimension reduction methods t-SNE, Isomap, LLE, kPCA and DM to the simulation data. Here, in the controlled environment we could clearly see that the PCA method was not able to identify all three true groups in the simulated data, but the ICS method, however, was. From the other tested methods, only t-SNE was able to recover all three subgroups, but all other four tested methods failed doing so. Some of them separated a single subgroup, but mixed the remaining two groups into a single large cluster. When the methods were then applied to a high-density genotype chicken data, the PCA method could not identify any subgroups. The ICS method clearly identified two subgroups consisting of 20, respective 10 samples, that share the same family background. Two (kPCA and LLE) of the five other methods, however, also detected the same subgroups in the real chicken data. The other three methods failed to identify any clear cluster structures.

In the scatterplot matrices some outlying observations could be identified by t-SNE (see Additional file 1), but not as evident as in the ICS case. A closer look at component 3 showed e.g., that some of the chicken with a value larger 25 are related but the most of them are unrelated. In terms of calculation times, the ICS needed around 0.2 s, whereas the t-SNE run took around three minutes. The other used methods needed at most only a few seconds for the calculation.

We considered also the red subgroup identified by ICS closer. It was superior in more than half of the available breeding values compared to the standard chicken population. Within the standard chicken population we could identify three subgroups that were either genetically close, intermediate or far away from this subgroup based on the most deviating chromosomal region. In addition, these three groups of the main population showed a directional trend in many traits, especially in the important production values P2 and P3. Also the blue subgroup is deviating in five breeding values from the main population, including the production values P3.

There were no other combinations with those parents in the data available so that no further investigations could be conducted to identify the reason for the subgroups to behave in such a different way. The biological explanation for the difference is beyond the scope of this paper.

The identification of three groups (main group and two subgroups) within the data is remarkable. As all the chicken originate from the same line, one would not assume any subpopulation structures and by applying a PCA to it, we did not identify any. ICS identified two subpopulations that were thereafter also seen to differ from the main population for some of the phenotypes for production traits.

Further, we could identify strongly deviating genetic regions between the subpopulations and the main group and followed exemplary up on the one that corresponds to the red subgroup. Within that, we calculated then the genetic distance of the remaining chicken to the identified subpopulation and could see that chicken genetically more similar with regard to the deviating region to the subpopulation also have better production values. Moreover, we could identify a directional relationship between the genetic similarity in that region and certain production values.

## Conclusion

We presented here an alternative dimension reduction method that is already used in other scientific fields, but that has not yet made its way to the genomic community. However, although ICS is superior over PCA in the current scenario, its purpose is not to replace PCA or any other dimension reduction method, but it is rather considered to be another tool in the dense genotype data analysis toolbox.

Its good results for both, the simulation and the real dataset encourage its use also for other genomic datasets to further evaluate its performance in a larger scale. Compared to other, modern dimension reduction methods, we saw that there is a large variation in the performance of each method, depending on the dataset. For our data, only ICS showed good results in the simulation as well as in the real data set, Isomap and Diffusion map had the weakest results for both setups. t-SNE only performed well in the simulation setup and LLE only for the real data.

## Additional file

Additional file 1 The supplemental material contains additional Figures. Included figure files are provided below. **Figure S1.** Scatterplot matrix of the unmixed simulation data. **Figure S2.** Scatterplot matrix of the rotated simulation data. **Figure S3.** Visualization of genomic regions that have been used for the analysis. **Figure S4.** Scatterplot with cluster labels of the k-means clustering for t-SNE, Isomap and the LLE. **Figure S5.** Scatterplot with cluster labels of the k-means clustering for Diffusion Maps and kernel PCA. **Figure S6.** Scatterplot matrix for t-SNE output with k=7 applied to the real chicken data. **Figure S7.** Scatterplot matrix for t-SNE output with k=2 applied to the real chicken data. **Figure S8.** Scatterplot matrix of the first 7 components of the LLE output applied to the real chicken data. **Figure S9.** Scatterplot matrix of the first 7 components of the Isomap output applied to the real chicken data. **Figure S10.** Scatterplot matrix of the first 7 components of the kPCA output applid to the real chicken data. **Figure S11.** Scatterplot matrix of the first 7 components of the DM output applied to the real chicken data. **Figure S12.** The values of the level of agreement across the regions of interest. **Figure S13.** The individual genetic distances between main population to the mode of the subpopulation. (PDF 2130 kb)

## Abbreviations

DM: Diffusion maps
ICA: Independent component analysis
ICS: Invariant coordinate selection
kPCA: kernel PCA
LDA: Linear discriminant analysis
LLE: Locally linear embedding
PCA: Principle component analysis
SNP: Single nucleotide polymorphism
t-SNE: t-Distributed stochastic neighbor embedding

## References

[1] Solovieff N, Hartley SW, Baldwin CT, Perls TT, Steinberg MH, Sebastiani P. Clustering by genetic ancestry using genome-wide snp data. BMC Genet. 2010; 11. doi:10.1186/1471-2156-11-108. 10.1186/1471-2156-11-108 PMC3018397 21143920

[2] Patterson N, Price AL, Reich D (2006). Population structure and eigenanalysis. PLoS Genet.

[3] Ma S, Dai Y (2010). Principal component analysis based methods in bioinformatics studies. Brief Bioinforma.

[4] Caussinus H, Ruiz A. Interesting Projections of Multidimensional Data by Means of Generalized Principal Component Analyses In: Momirović K, Mildner V, editors. Compstat: Proceedings in Computational Statistics, 9th Symposium held at Dubrovnik, Yugoslavia, 1990. Heidelberg: Physica-Verlag HD: 1990. p. 121–6. doi:10.1007/978-3-642-50096-1_19.

[5] Tyler DE, Critchley F, Dümbgen L, Oja H (2009). Invariant co-ordinate selection. J R Stat Soc Series B.

[6] Vidal R, Ma Y, Sastry SS (2016). Generalized Principal Component Analysis.

[7] Musial JP, Hüsler F, Sütterlin M, Neuhaus C, Wunderle S (2014). Daytime low stratiform cloud detection on avhrr imagery. Remote Sensing.

[8] Musial JP, Hüsler F, Sütterlin M, Neuhaus C, Wunderle S (2014). Probabilistic approach to cloud and snow detection on advanced very high resolution radiometer (avhrr) imagery. Atmos Meas Tech.

[9] Bookstein FL, Mitteroecker P (2013). Comparing covariance matrices by relative eigenanalysis, with applications to organismal biology. Evol Biol.

[10] van der Maaten LJP, Hinton GE (2008). Visualizing high-dimensional data using t-sne. J Mach Learn Res.

[11] Tenenbaum JB, de Silva V, Langford JC (2000). A global geometric framework for nonlinear dimensionality reduction. Science.

[12] Roweis S, Saul L (2000). Nonlinear dimensionality reduction by locally linear embedding. Science.

[13] Schölkopf B, Smola A, Müller KR (1998). Nonlinear component analysis as a kernel eigenvalue problem. Neural Comput.

[14] Coifman RR, Lafon S, Lee AB, Maggioni M, Nadler B, Warner F, Zucker SW (2005). Geometric diffusions as a tool for harmonic analysis and structure definition of data: Diffusion maps. PNAS.

[15] Tuiskula-Haavisto M, Honkatukia M, Preisinger R, Schmutz M, de Koning DJ, Wei WH, Vilkki J (2011). Quantitative trait loci affecting eggshell traits in an f2 population. Animal Genet.

[16] Honkatukia M, Tuiskula-Haavisto M, Arango J, Tabell J, Schmutz M, Preisinger R, Vilkki J (2013). Qtl mapping of egg albumen quality in egg layers. Genet Sel Evol.

[17] Nordhausen K, Tyler DE. A cautionary note on robust covariance plug-in methods. Biometrika. 2015. doi:10.1093/biomet/asv022.

[18] Nordhausen K, Oja H, Ollila E. Multivariate Models and the First Four Moments. Singapore: World Scientific; 2011, pp. 267–87. doi:10.1142/9789814340564_0016.

[19] Nordhausen K, Oja H, Tyler DE (2008). Tools for exploring multivariate data: The package ICS. J Stat Softw.

[20] Miettinen J, Taskinen S, Nordhausen K, Oja H (2015). Fourth moments and independent component analysis. Statist Sci.

[21] Tapio M, Tapio I, Grislis Z, Holm LE, Jeppsson S, Kantanen J, Miceikiene I, Olsaker I, Viinalass H, Eythorsdottir E (2005). Native breeds demonstrate high contributions to the molecular variation in northern european sheep. Mol Ecol.

[22] Tyler DE (2010). A note on multivariate location and scatter statistics for sparse data sets. Stat Probab Lett.

[23] Oja H, Nordhausen K, El-Shaarawi A-H, Piegorsch W (2012). Independent Component Analysis. Encyclopedia of Environmetrics.

[24] Rand WM (1971). Objective criteria for the evaluation of clustering methods. J Am Stat Assoc.

[25] Fischer D, Oja H, Schleutker J, Sen PK, Wahlfors T (2014). Generalized Mann-Whitney type tests for microarray experiments. Scand J Stat.

[26] Fischer D, Oja H. Mann-Whitney type tests for microarray experiments: The R package gMWT. J Stat Softw. 2015; 65(1):1–19. doi:10.18637/jss.v065.i09.

[27] Donaldson J. Tsne: T-Distributed Stochastic Neighbor Embedding for R (t-SNE). 2016. R package version 0.1-3. http://CRAN.R-project.org/package=tsne. Accessed 30 Nov 2016.

[28] Bartenhagen C. RDRToolbox: A Package for Nonlinear Dimension Reduction with Isomap and LLE. 2014. R package version 1.20.0. https://www.bioconductor.org/packages/release/bioc/html/RDRToolbox.html. Accessed 30 Nov 2016.

[29] Diedrich H, Abel M. Lle: Locally Linear Embedding. 2012. R package version 1.1. http://CRAN.R-project.org/package=lle. Accessed 30 Nov 2016.

[30] Karatzoglou A, Smola A, Hornik K, Zeileis A (2004). kernlab – an S4 package for kernel methods in R. J Stat Softw.

[31] Angerer P, Haghverdi L, Büttner M, Theis FJ, Marr C, Buettner F. destiny – diffusion maps for large-scale single-cell data in R. Bioinformatics. 2015. doi:10.1093/bioinformatics/btv715.http://bioinformatics.oxfordjournals.org/content/early/2015/12/13/bioinformatics.btv715.full.pdf+html. 10.1093/bioinformatics/btv715 26668002

[32] Zheng X, Levine D, Shen J, Gogarten S, Laurie C, Weir B (2012). A high-performance computing toolset for relatedness and principal component analysis of snp data. Bioinformatics.

