# Supplementary material for: Subgroup detection in genotype data using invariant coordinate selection
Source: BMC Bioinformatics. 2017 Mar 16;18:173. doi: 10.1186/s12859-017-1589-9 (PMC5356247; doi:10.1186/s12859-017-1589-9)
Supplement: Additional file 1 — The supplemental material contains additional Figures. Included figure files are provided below. Figure S1. Scatterplot matrix of the unmixed simulation data. Figure S2. Scatterplot matrix of the rotated simulation data. Figure S3. Visualization of genomic regions that have been used for the analysis. Figure S4. Scatterplot with cluster labels of the k-means clustering for t-SNE, Isomap and the LLE. Figure S5. Scatterplot with cluster labels of the k-means clustering for Diffusion Maps and kernel PCA. Figure S6. Scatterplot matrix for t-SNE output with k=7 applied to the real chicken data. Figure S7. Scatterplot matrix for t-SNE output with k=2 applied to the real chicken data. Figure S8. Scatterplot matrix of the first 7 components of the LLE output applied to the real chicken data. Figure S9. Scatterplot matrix of the first 7 components of the Isomap output applied to the real chicken data. Figure S10. Scatterplot matrix of the first 7 components of the kPCA output applid to the real chicken data. Figure S11. Scatterplot matrix of the first 7 components of the DM output applied to the real chicken data. Figure S12. The values of the level of agreement across the regions of interest. Figure S13. The individual genetic distances between main population to the mode of the subpopulation. (PDF 2130 kb) [file 12859_2017_1589_MOESM1_ESM.pdf]

Supplemental material: Subgroup Detection in Genotype Data using Invariant Coordinate Selection

D.Fischer M. Honkatukia M. Tuiskula-Haavisto
K. Nordhausen D. Cavero R. Preisinger J. Vilkki

February 27, 2017

1 Supplemental material

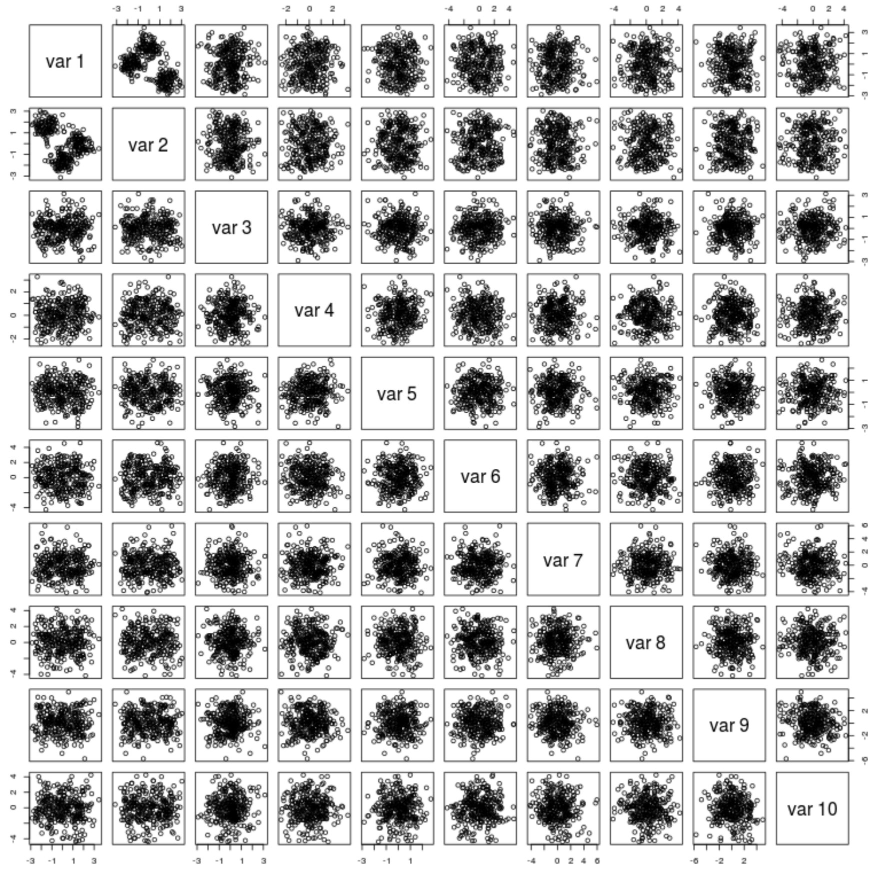

Figure S1: Scatterplot matrix of the unmixed simulation data.

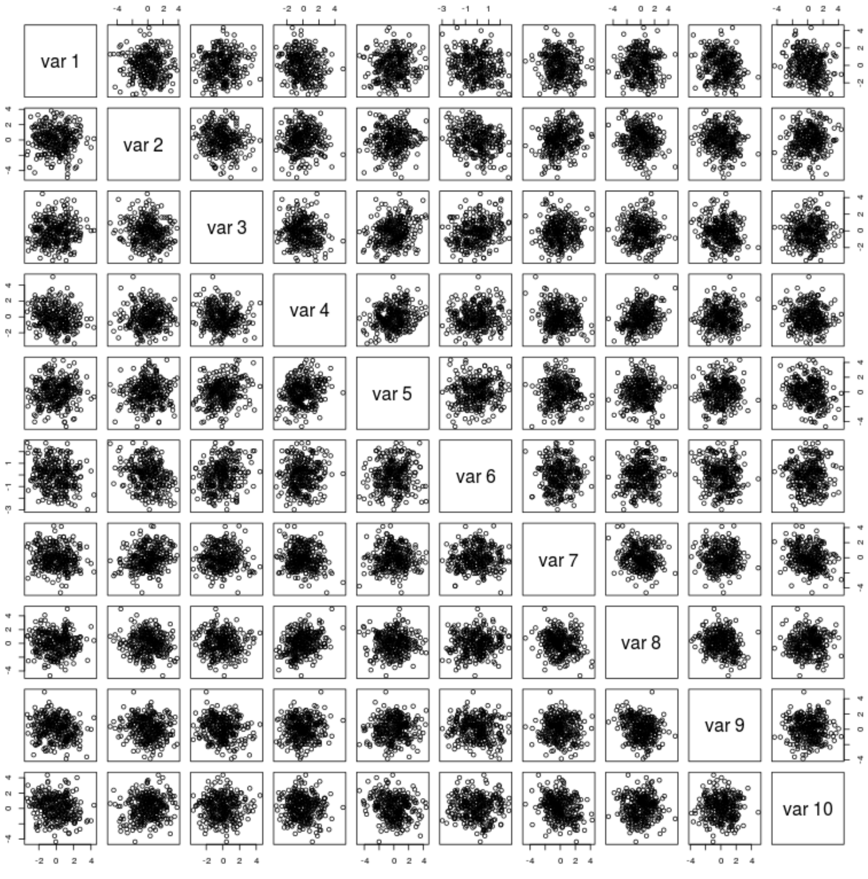

Figure S2: Scatterplot matrix of the rotated simulation data.

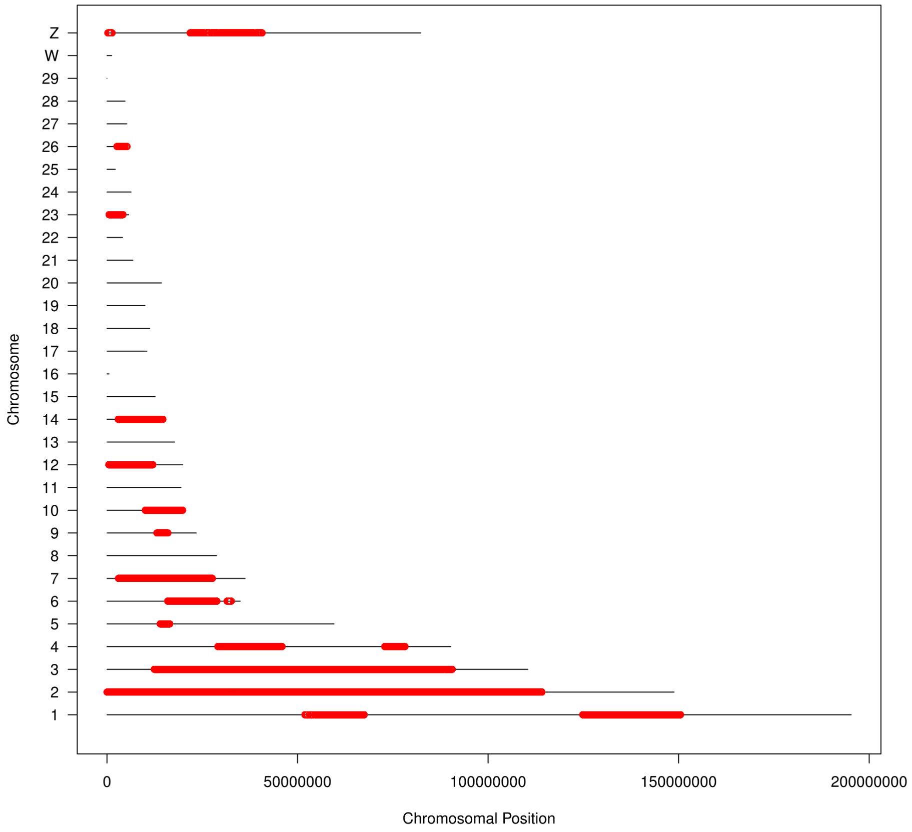

Figure S3: The red bars indicate the locations of the genomic regions that have been used for the analysis.

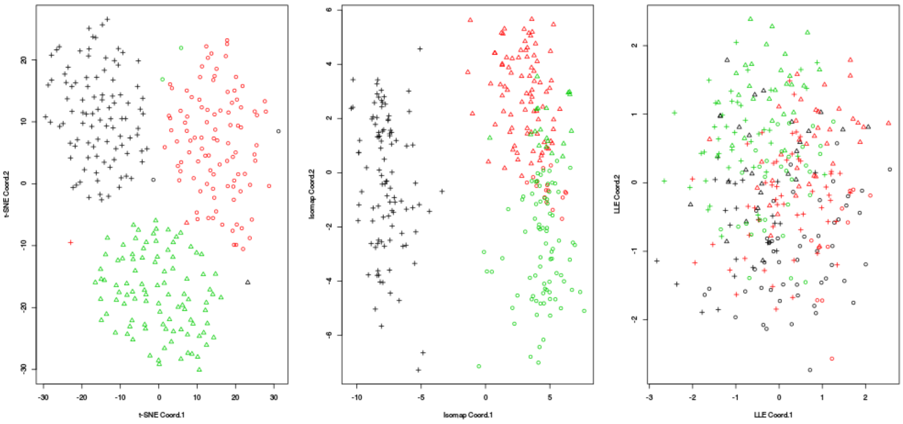

Figure S4: Cluster labels of the k-means clustering for t-SNE (left), Isomap (middle) and the LLE (right). The true class labels are colored accordingly and the k-means classification is represented with different symbols.

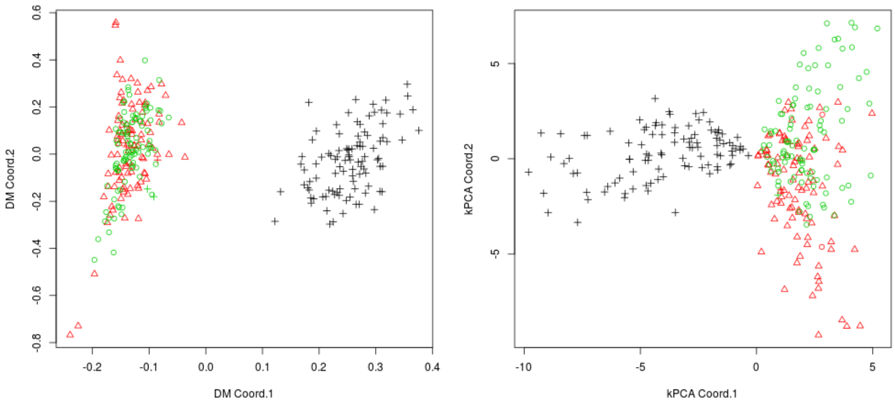

Figure S5: Cluster labels of the k-means clustering for Diffusion Maps (left) and kernel PCA (right). The true class labels are colored accordingly and the k-means classification is represented with different symbols.

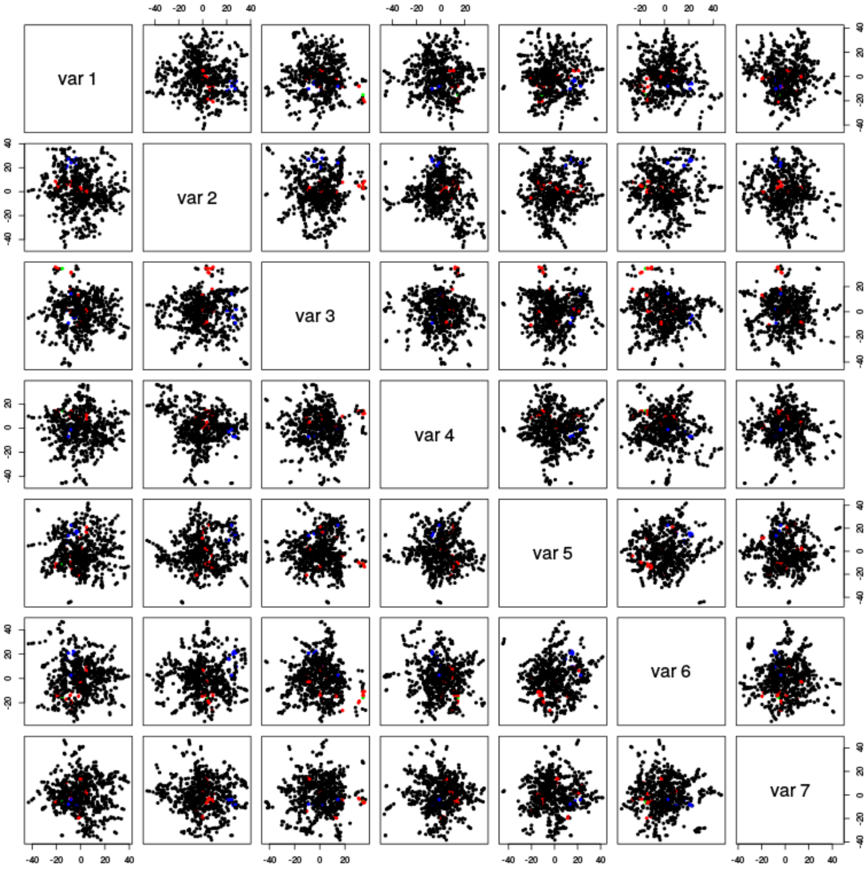

Figure S6: Scatterplot matrix for t-SNE output with k=7 applied to the real chicken data. The subgroups identified by ICS are highlighed in the red/blue.

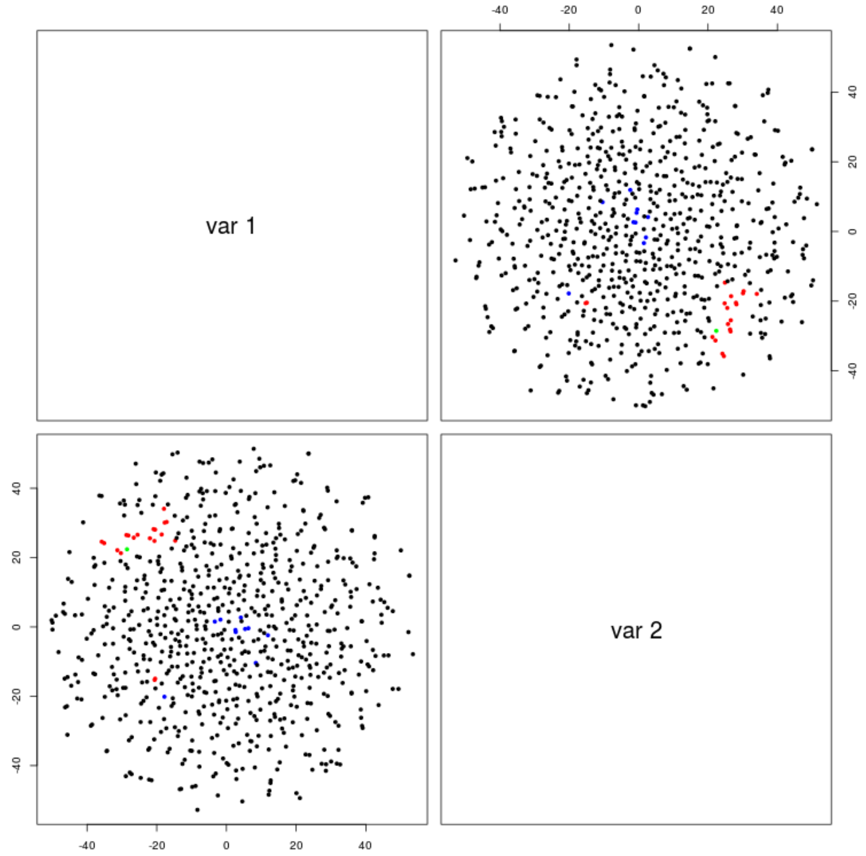

Figure S7: Scatterplot matrix for t-SNE output with k=2 applied to the real chicken data. The subgroups identified by ICS are highlighed in the red/blue.

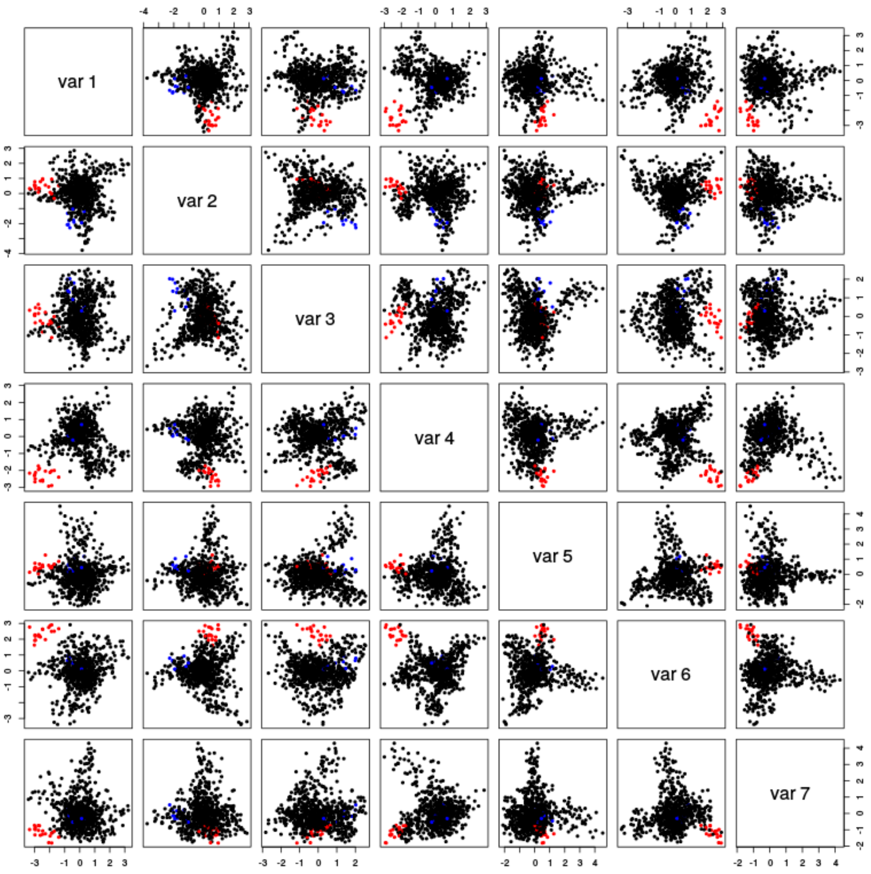

Figure S8: Scatterplot matrix of the first 7 components of the LLE output applied to the real chicken data. The subgroups identified by ICS are highlighed in the red/blue.

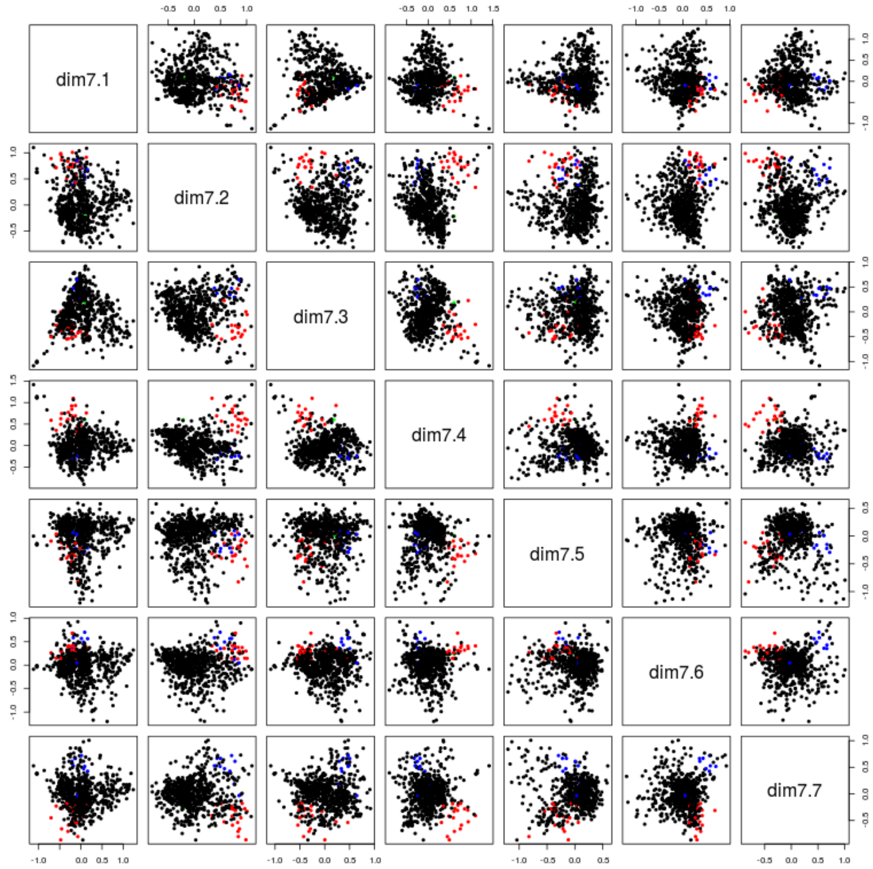

Figure S9: Scatterplot matrix of the first 7 components of the Isomap output applied to the real chicken data. The subgroups identified by ICS are highlighed in the red/blue.

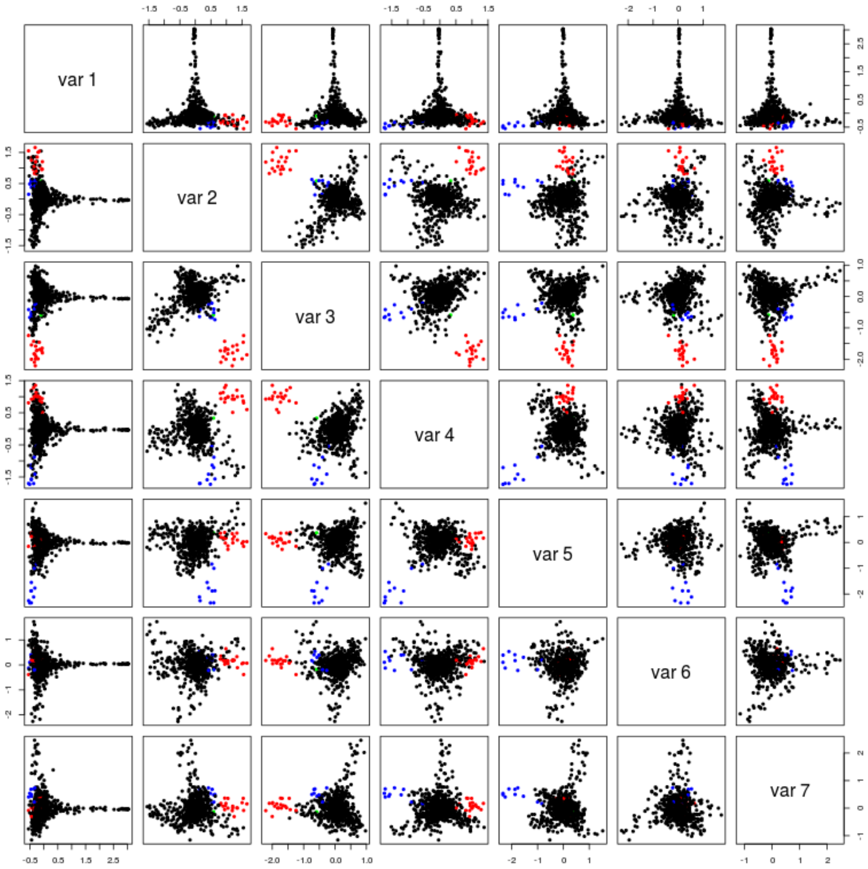

Figure S10: Scatterplot matrix of the first 7 components of the kPCA output applid to the real chicken data. The subgroups identified by ICS are highlighed in the red/blue.

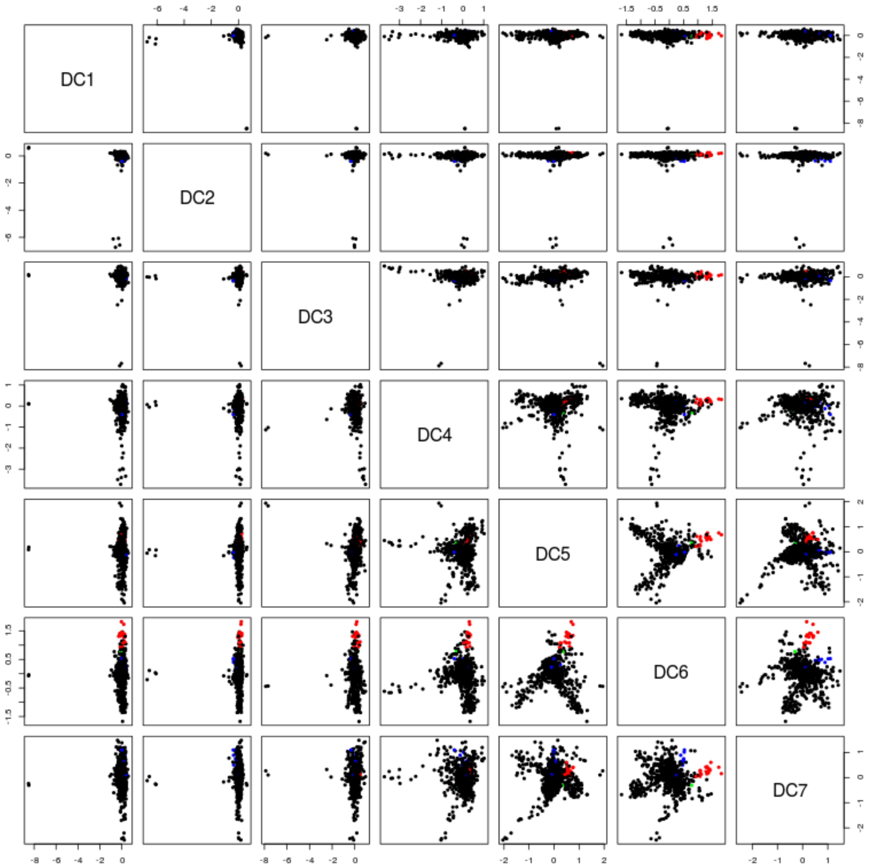

Figure S11: Scatterplot matrix of the first 7 components of the DM output applied to the real chicken data. The subgroups identified by ICS are highlighed in the red/blue.

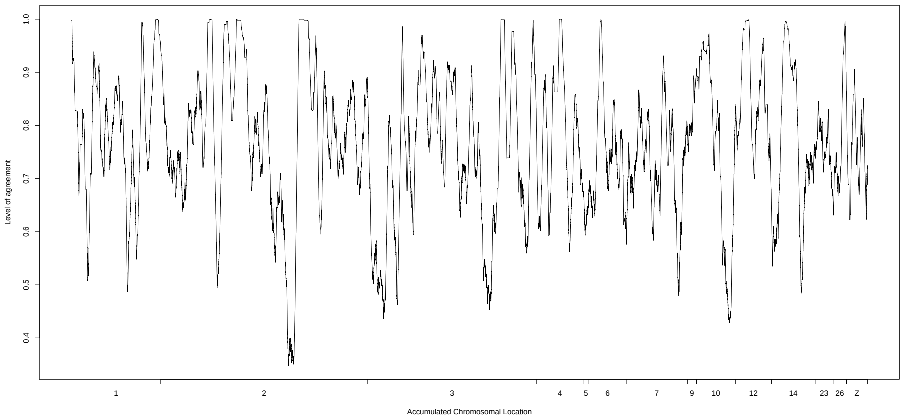

Figure S12: The values of the level of agreement across the regions of interest.

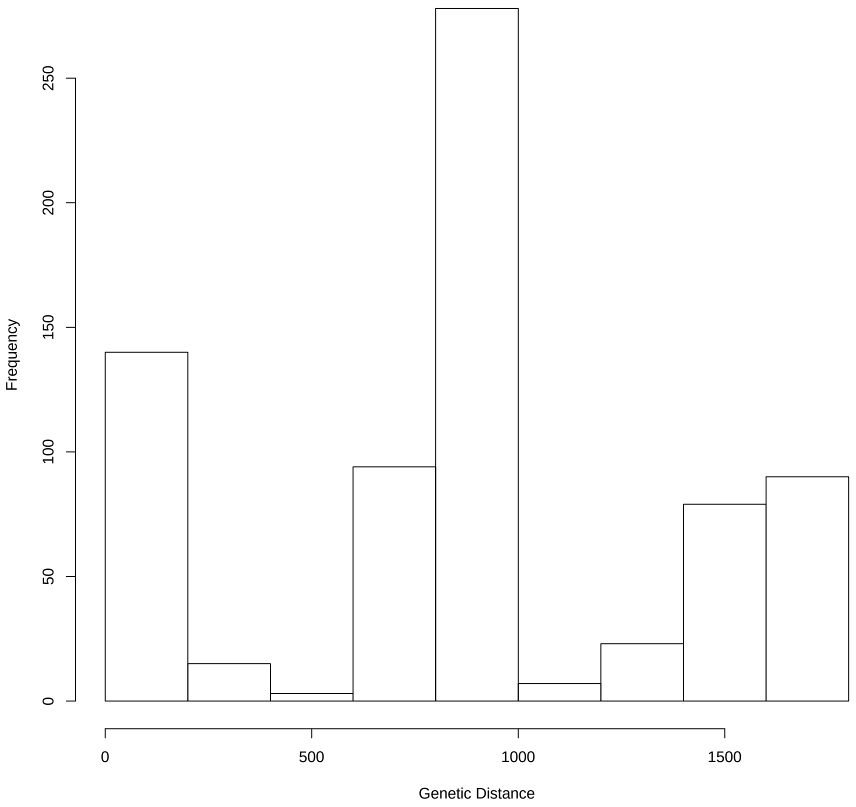

Figure S13: The individual genetic distances between main population to the mode of the subpopulation.
